# Ethics as Our Guide

**DOI:** 10.1371/journal.pbio.0020170

**Published:** 2004-06-15

**Authors:** Michael Cook

## Abstract

In response to the Blackburn and Rowley essay on the President's Council on Bioethics, several thought-provoking opinions on ethical challenges in biomedical research are expressed by prominent stakeholders

Blackburn and Rowley's (2004) criticism of a report on embryonic stem cell research from the President's Council on Bioethics (2004) is puzzling. Where is the bioethics? The nub of their complaint is that some details of the report have been partisan and have distorted ‘the potential of biomedical research and the motivation of some of its researchers’. No doubt their quibbles are well-founded, as every committee report is a compromise.

However, it does not follow that if the benefits of embryo stem cell research had been presented more persuasively and in greater detail, then the case for ‘non-commercial, federal, peer-reviewed funding’ would be unassailable. Such a view appears to be based squarely on a utilitarian view of the moral status of embryos: that the good flowing from destructive research outweighs the evil of embryo destruction. Far from being a neutral scientific analysis, this expresses a commitment to the proposition that biomedical progress is more important than the defence of human life.

If twentieth century philosophy of science has taught us anything, it is that the aspiration to pure scientific objectivity is a dangerous illusion. Research programs always embody philosophical and moral assumptions that must be openly defended. If Blackburn and Rowley want government support for embryo stem cell research, they must justify their bioethical approach and not hide behind a smokescreen of indignation over Blackburn's unwilling departure from the Council.
